# Does Consuming Fresh Ultraviolet Light-Exposed Mushrooms Offset the Seasonal Decline in Serum Total 25OHD in Adults Classified as Overweight and Class I Obese? Results from a Randomized Controlled Trial

**DOI:** 10.3390/foods15091572

**Published:** 2026-05-02

**Authors:** Luz M. Comboni, Emily S. Glover, Skye C. Napolitano, James C. Fleet, Dan Foti, Matthew R. Olson, Wayne W. Campbell

**Affiliations:** 1 Department of Nutrition Science, Purdue University, West Lafayette, IN 47907, USA; lcomboni@purdue.edu (L.M.C.);; 2 Department of Psychological Sciences, Purdue University, West Lafayette, IN 47907, USA; 3 Department of Nutritional Sciences, The University of Texas at Austin, Austin, TX 78712, USA; james.fleet@austin.utexas.edu; 4 Department of Biological Sciences, Purdue University, West Lafayette, IN 47907, USA

**Keywords:** vitamin D status, *Agaricus bisporus*, cremini mushrooms, ergocalciferol

## Abstract

We assessed whether consuming UV light-exposed mushrooms (UVMs) would offset the seasonal decline in circulating total 25-hydroxyvitamin D (25OHD), an index of vitamin D status. During late fall and winter, 41 adults (19 M/22 F, age 43 ± 11 y, BMI 29.8 ± 5.9 kg/m^2^, mean ± SD) were randomized to consume either 84 g of fresh *Agaricus bisporus* twice/d (produced to contain 400 IU of vitamin D_2_/serving; 800 IU/d total) or 1 tsp of breadcrumbs twice/d (Control) while continuing to consume their self-chosen diets. At baseline and week 6, fasting serum 25OHD_2_, 25OHD_3_, and total 25OHD were measured. Mushrooms were sampled weekly and vitamin D_2_ content measured. From the intent-to-treat analysis (Mushroom group, *n* = 20, and Control group, *n* = 21), 25OHD_2_ increased and 25OHD_3_ decreased in the Mushroom group over winter months compared to Control with no differences in the decrease in total 25OHD between groups. Unexpectedly, only 67% of the UVMs contained vitamin D_2_. Post hoc subgroup assessment indicated that participants consuming UVMs (*n* = 11) had increased 25OHD_2_ and a greater decline in 25OHD_3_ compared to subgroups consuming mushrooms without vitamin D_2_ (*n* = 9) and Control (*n* = 21), with no differences in the decrease in total 25OHD among subgroups. Consuming UVMs increased serum 25OHD_2_ but did not prevent a seasonal decline in vitamin D status due to a greater decrease in 25OHD_3_.

## 1. Introduction

Despite scientific advancements in supplements and food fortification, vitamin D deficiency remains a global public health concern, affecting nearly 1 billion people worldwide [1]. In the United States, 24.6% and 41% of adults are vitamin D deficient (<12 ng/mL or <30 nmol/L) and insufficient (12 to <20 ng/mL or 30 to <50 nmol/L), respectively [2]. Individuals classified as overweight or obese (BMI ≥ 25 kg/m^2^) are at greater risk for vitamin D insufficiency and deficiency as greater body fat mass and BMI predict lower serum total 25-hydroxyvitamin D (25OHD) concentrations [3,4]. This population classified as overweight and obese living in high northern (≥40° N) or southern latitudes (≥40° S) are at even greater risk for insufficiency and deficiency during winter months due to the lack of sunlight of appropriate wavelength for endogenous synthesis of vitamin D_3_ [5]. Thus, additional sources of vitamin D are important to maintain vitamin D status during winter months.

Dietary sources of vitamin D are few and include fatty fish, eggs, and fortified milks [6]. Interestingly, mushrooms irradiated with ultraviolet B (UVB) light typically contain about 9.2 μg (366 IU) of vitamin D_2_ per serving (84 g) [6]. Product labeling of commercially UVB-irradiated mushrooms indicates mushrooms contain at minimum 400 IU of vitamin D_2_ per serving [7]. The Recommended Dietary Allowance (RDA) for vitamin D for healthy people aged 1–70 y is 15 μg/d (600 IU/d) and for those aged > 70 y, 20 μg/d (800 IU/d) [8]. Specific guidelines for the intake of vitamin D among people with overweight and obesity have not been established [6,9]. Given that UV light-exposed mushrooms (UVMs) are a significant source of vitamin D_2_ [10], regular consumption of UVMs can contribute to meeting the RDA for vitamin D.

Research assessing the effects of UVMs to negate a decline in total 25OHD during winter months is inconsistent. These studies used fresh or powdered UVMs with a median vitamin D_2_ dose of 25 μg/d (1000 IU/d). The results range from positive (increase [11,12,13,14] or negating the decline [15,16,17,18,19] in total 25OHD) to neutral (no influence on the seasonal decline [20]). Importantly, only one study [15] assessed this outcome among people classified as overweight and class I obese. Thus, the effectiveness of fresh UVM consumption to maintain or increase vitamin D status during winter months in this population is not clearly established. The purpose of our research was to assess the effectiveness of UVMs versus a non-mushroom, non-vitamin D control on changes in vitamin D status during winter months in adults classified as overweight and class I obese. To our knowledge, this study is the first to compare UVMs with a non-mushroom, non-vitamin D control in this population. We hypothesized that consuming UVMs, compared to consuming the control, would increase serum 25OHD_2_ and offset the seasonal decline in serum total 25OHD, an important index of vitamin D status.

## 2. Materials and Methods

### 2.1. Ethics

The study protocol was approved by the Purdue University Institutional Review Board (IRB 2022-750; initial date approved: 15 September 2022) and registered in the public registry clinicaltrials.gov prior to participant recruitment (https://clinicaltrials.gov/study/NCT05559112; initial date submitted: 26 September 2022). Informed consent was obtained from all participants before the study commenced. Participants received monetary compensation for their time.

### 2.2. Participant Recruitment and Eligibility Criteria

Volunteers were recruited from the Greater West Lafayette and Lafayette, IN, region (40.4° N, 86.9° W) in the United States of America using various recruitment strategies. We hung physical fliers on Purdue University’s campus and in the greater community, posted an announcement on Purdue University’s daily newsletter, and advertised on social media by contracting with Trialfacts, a company specializing in participant recruitment (Melbourne, Australia).

Eligibility criteria are published [21] and are listed in Supplementary Material S1 Table S1. Briefly, male and female volunteers aged 30–69 years classified as overweight or class I obese (25.0–34.9 kg/m^2^) who were not acutely ill, nonsmoking, nondiabetic, and females who were not pregnant or lactating were eligible for a health screening. Participants were required to be willing and able to consume mushrooms and travel to testing facilities, not take vitamin D supplements, and refrain from vacationing in sunny locations or using tanning beds. Individuals taking high doses of vitamin D (>3000 IU/d) were not eligible for the study, and individuals taking <3000 IU/d but greater than the RDA (600 IU/d) were instructed to stop taking the supplements at least four weeks prior to baseline testing. Participants maintained their habitual diet and levels of physical activity during the intervention.

Participants completed a health screening at our research facility, and their enrollment in the study was contingent on our study physician’s evaluation of their medical history and bloodwork results.

### 2.3. Experimental Design

**Figure 1:**
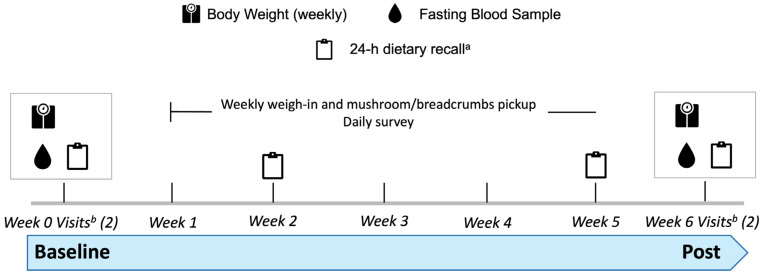
Diagram of the 6-week timeline and schedule of commitment for participants. ^a^ Participants filled out the Automated Self-Administered 24 h Dietary Assessment Tool (NCI/NIH) on random week and weekend days during weeks 0 (baseline), 2, and 5. ^b^ At baseline (week 0) and post (week 6), we performed repeated testing (two visits, ≥48 h apart). Diagram adapted from Glover et al. 2025 [21].

We used a parallel, randomized, controlled study design. During late October to early April (2022–2023 and 2023–2024), 41 middle-aged and older adults without diagnosed morbidities completed a 6-week trial (Figure 1). While continuing to consume their self-chosen diets, participants were randomized to consume either 84 g of fresh *Agaricus bisporus* (cremini mushrooms; produced to contain 400 IU of vitamin D_2_) twice/d (800 IU/d) or 1 tsp of commercial breadcrumbs twice/d (Control). Participants were randomized using Permuted Block Randomization with random block constellation (RPBR) [22,23]. The primary outcome was vitamin D status and included measurements of serum 25OHD_2_ and 25OHD_3_ and summed these for total 25OHD. Secondary outcomes were immune-related, which we will report in a separate manuscript.

### 2.4. Dietary Intervention and Assessment

Weekly, participants picked up fresh mushrooms or breadcrumbs from our research facility. Participants consuming mushrooms picked up their seven-day fresh mushroom supply on the day of or no later than 48 h after delivery of the mushroom shipment. The commercially produced and package-labeled mushrooms were donated by Monterey Mushrooms farms in Princeton, IL, and Madisonville, TX, USA. Participants consuming breadcrumbs picked up their seven-day breadcrumb supply portioned out into seven plastic containers labeled with the date they were packaged. On pick-up day, participants weighed themselves wearing light clothing and received instructions and reminders on storage, portioning, handling, and recording their consumption of mushrooms or breadcrumbs. Participants were instructed to store mushrooms in their original packaging within the brown paper bag provided and to avoid handling until time of consumption. Upon consumption, participants were to weigh 84 g raw weight of mushrooms using the weighing scale provided, to rinse mushrooms briefly, and pat dry. Cooking methods included raw, sauteed, or boiled. Participants were discouraged from baking mushrooms in the oven. Those consuming breadcrumbs were instructed to store the breadcrumbs in a cool and dry location away from direct sunlight. Upon consumption, participants were to measure 1 teaspoon of breadcrumbs and consume them with their meal. All participants were instructed and reminded weekly to complete a daily online survey.

Data from a daily online survey was used to assess self-reported adherence to the assigned intervention. In this survey, participants were asked whether they consumed their required daily amounts of mushrooms or control food and were prompted to report any factors that might affect study outcomes (e.g., illness, gastrointestinal symptoms, medication changes, etc.). Adherence to consuming the UVMs could not be assessed objectively with serum 25OHD_2_ concentrations because analysis of the UVMs revealed that 33% of mushrooms did not contain vitamin D_2_ (<6 ng/g). We do not know why some of the mushrooms did not contain vitamin D_2_.

The automated Self-Administered 24 h Dietary Assessment Tool, ASA24, version 2022, developed by the National Cancer Institute of the National Institutes of Health, was used to estimate their daily food consumption. Each participant completed one assessment at baseline and the remaining two assessments on nonconsecutive random week and weekend days during the intervention. Dietary intake data were used to calculate participants’ Healthy Eating Index (HEI) scores, and HEI scores were used to assess diet quality and consistency throughout the intervention. A HEI score can range from zero to 100 arbitrary units, with 100 au ideal, and is a metric used to estimate a person’s overall diet quality based on the Dietary Guidelines for Americans recommendations (HEI-2015 [24]).

### 2.5. UV-Exposure, Sampling, and Analysis of Mushrooms

The specific steps the farms in Princeton, IL, and Madisonville, TX, USA, used to enrich mushrooms are proprietary. However, the general methodology is published [7]. Briefly, mushrooms are harvested, washed, placed on a conveyor belt under UVB light bulbs, sliced, cooled, and packaged. To our knowledge, both farms followed the same methodology of UV light exposure.

Weekly, on the day a mushroom shipment arrived, research staff randomly collected two mushroom samples (each 84 g). Each fresh mushroom sample was double-bagged in freezer-grade reclosable plastic bags and frozen at −4 °C. Three frozen mushroom samples (from three different weeks) were sent to Heartland Assays, Ames, IA, USA, for vitamin D analysis during the study intervention. The remaining samples were batched and sent for vitamin D analysis at the study’s conclusion. A method for quantitating vitamin D_2_ and D_3_ in complex matrices was validated in the laboratory at Heartland Assays. Samples were spiked with internal standard (d3-VitaminD_3_) and saponified with methanolic potassium hydroxide. Samples and controls were liquid–liquid extracted using hexane and methylene chloride before going through a solid phase extraction step using an Agilent Bond Elut 1.0 g Silica column. One straight phase HPLC (Agilent 1100) was used to purify vitamin D_2_ and D_3_ analytes; the attached column was an Agilent Zorbax Sil 5 um 9.4 × 250 mm. Final quantitation was performed on an Agilent 1290 LC/MS/MS with an attached Agilent Poroshell 120 EC C18 2.7 um 2.1 × 50 mm column. Vitamin D_3_ content was below the level of quantitation (<0.5 ng/g) for all samples. Therefore, we report content of vitamin D_2_ only.

### 2.6. Clinical Assessments and Blood Processing

During baseline and week-6 testing days, participants arrived at the Purdue University research facility after fasting for 8 to 10 h overnight. We measured bodyweight at both timepoints. Participants then rested in a seated position for 15 min before a trained phlebotomist collected a blood sample from an antecubital vein into vacutainers. The vacutainers, containing a clot-activating gel, were maintained at room temperature for 15–30 min or until clotting occurred and subsequently centrifuged at 4000× *g* at 4 °C for 15 min. Serum samples were stored at −80 °C until they were shipped to Heartland Assays, Ames, IA, for 25OHD_2_ and 25OHD_3_ analysis using liquid chromatography–tandem mass spectrometry (LC/MS/MS). The protocol is published [25,26,27,28,29].

### 2.7. Statistical Analysis

Intent-to-treat (ITT) analyses were performed to assess differences in outcome variables. Because 33% of mushrooms contained amounts <6 ng/g of vitamin D_2_, we could not use serum 25OHD_2_ concentrations to objectively measure adherence to the intervention. Thus, we could not determine if the lack of 25OHD_2_ in the serum of some participants in the Mushroom group was due to lack of adherence to the intervention or lack of vitamin D_2_ in the mushrooms they consumed. To compensate, we performed additional sub-analyses based on post hoc grouping of participants according to serum 25OHD_2_ concentrations at week 6. In the first sub-analysis, we assessed the changes in 25OHD_2_, 25OHD_3_, and total 25OHD among participants who had detectable concentrations of serum 25OHD_2_ (>1.5 ng/mL) at week 6 (Mushroom + VitD_2_, *n* = 11) compared to the changes among all other participants (i.e., those who did not have vitamin D_2_ at week 6, *n* = 11, combined with those in the Control group, Control-VitD_2_, *n* = 30). In the second sub-analysis, we compared three groups: those with 25OHD_2_ (Mushroom + VitD_2_, *n* = 11) at week 6 vs. no 25OHD_2_ at week 6 (Mushroom-VitD2, *n* = 9) vs. ITT Control (*n* = 21).

We employed a repeated measures ANCOVA model to assess the effects of time, intervention group, and their interaction on serum 25OHD_2_, 25OHD_3_, and total 25OHD concentrations. Pairwise comparisons were used to identify groups with a significant difference. The final model included sex as a covariate since sex explained variation in the outcome of interest. We tested other covariates, BMI and age, and found they did not explain variation in the outcome of interest. Thus, BMI and age covariates were dropped from the final model. The primary conclusion of this research is based on the ITT analysis.

We corrected for likelihood of type-I error from multiple comparisons using a False Discovery Rate set at 5% significance [30]. Of note, two participants (*n* = 2) dropped out after baseline testing and were excluded from all analyses. Thus, we used data from 41 participants for the statistical analyses, which were performed using IBM SPSS Statistics (version 29.0.2.0, IBM Corp., Armonk, NY, USA).

No studies with the same study design were available at the time we designed this study. Cost considerations from repeated testing required that we limit the number of participants per group to *n* = 20. Retrospectively, based on our hypothesis and using a sample size of *n* = 20 per group and at α = 0.05 and 1 − β = 0.8, we can detect a difference of 3.3 ng/mL in serum total 25OHD. We describe our rationale of calculating power in Supplementary Material S2. We also calculated effect sizes using Cohen’s d [31] (shown in Supplementary Material S1 Table S2).

## 3. Results

### 3.1. Participants

**Figure 2:**
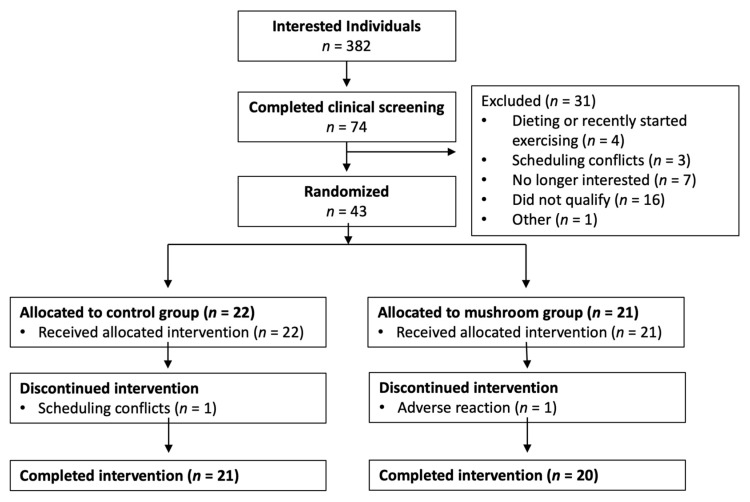
CONSORT diagram of participant flow.

Forty-one participants (19 males and 22 females, age 43 ± 11 y; BMI 29.8 ± 5.9 kg/m^2^, mean ± SD) without diagnosed morbidities completed this parallel, randomized controlled trial. Study coordinators were in contact with 382 interested individuals during the clinical testing period (late autumn to late winter 2022–2023 and 2023–2024) (Figure 2). Forty-three people were randomized to either Mushroom (*n* = 21) or Control (*n* = 22) groups. Forty-one participants completed the intervention. There were no differences between groups at baseline for demographic or clinical characteristics (Table 1).

### 3.2. Adherence to Consuming the Mushrooms/Control and Vitamin D_2_ Content in the Mushrooms

Self-reported adherence to the intervention was calculated based on the number of days participants reported adherence or non-adherence compared to the number of days they were in the study. Overall adherence to the assigned intervention was high, averaging 97.2% across all participants, with 96.6% adherence in the Mushroom group and 97.7% in the Control group. Our initial plan was to confirm adherence to the mushroom intervention by documenting the appearance of vitamin D_2_ metabolites in the serum of participants. However, self-reported adherence could not be confirmed objectively with serum 25OHD_2_ concentrations since 33% of the mushrooms did not contain the expected 119 ng/g of vitamin D_2_ (3.4 ± 2.5 ng/g, mean ± SD; range = 0.9–7.5 ng/g). During the six-week intervention period, the overall average of vitamin D_2_ in the mushrooms (intake proxy) was 169 ± 119 ng/g, which equates to 566 IU/84 g serving, and 1120 IU/d (Supplementary Material S3 Table S1).

### 3.3. Dietary Intake

Participants completed daily food records using the Automated Self-Administered 24 h Dietary Assessment Tool, once at baseline and twice during the intervention. The three food records were used to estimate energy intake (kcal/d), macronutrient composition (% energy), and dietary vitamin D content (μg and IU/d) in participants’ meals (Table 2). Mean HEI scores (arbitrary units, au, range 0–100 [24]) were calculated separately for each group and in combination at baseline, throughout the intervention, and overall (Table 3).

### 3.4. Intent-to-Treat (ITT; Planned Analysis)

**Figure 3:**
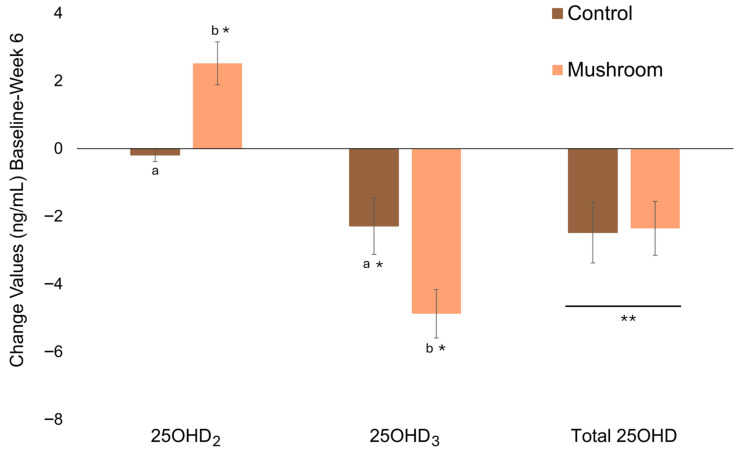
Intent-to-treat: Change values (ng/mL; baseline to week 6) of serum 25OHD_2_, 25OHD_3_, and total 25OHD for participants consuming mushrooms or control. * Denotes significant change from baseline. Different letters (a, b) denote significant differences in the change values between groups. ** Denotes main effect of time in the absence of an interaction effect.

Based on the ITT analysis (Mushroom, *n* = 20, vs. Control, *n* = 21), consuming mushrooms intended to contain vitamin D_2_ increased 25OHD_2_ (Time × Group, *p* < 0.001; large effect size, Cohen’s d = 1.31) but did not maintain total 25OHD (Time × Group, *p* = 0.910; small effect size, Cohen’s d = −0.03) due to greater decreases in 25OHD_3_ (Time × Group, *p* = 0.026; medium effect size, Cohen’s d = −0.73). The increase in 25OHD_2_ in the Mushroom group (2.4 ± 0.5, mean ± SE) differed from Control (0.1 ± 0.5, *p* < 0.001) and the decrease in 25OHD_3_ was greater in the Mushroom group (−4.9 ± 0.8 ng/mL, mean ± SE) compared to Control (−2.3 ± 0.8 ng/mL, *p* = 0.012). Total 25OHD decreased independently of mushroom intake (main effect of time, *p* = 0.008). The interaction (change values) and main effects are depicted in Figure 3 and summarized in Supplementary Material S1 Table S3.

### 3.5. Post Hoc Analysis Based on Measured Serum 25OHD_2_ Concentrations

At week 6, sera from nine participants in the Mushroom group showed vitamin D_2_ concentrations below the limit of quantitation (<1.5 ng/mL). Evaluation of vitamin D_2_ content in the mushrooms supported that this was likely an issue with the mushrooms (Supplementary Material S3). To scientifically address the lack of vitamin D_2_ in the mushrooms supplied to some participants, we conducted a post hoc analysis where we grouped these nine participants with the ITT Control group (Control-VitD_2_, *n* = 30) and compared them to participants with detectable serum 25OHD_2_ concentrations (>1.5 ng/mL) at week 6 (Mushroom + VitD_2_, *n* = 11).

**Figure 4:**
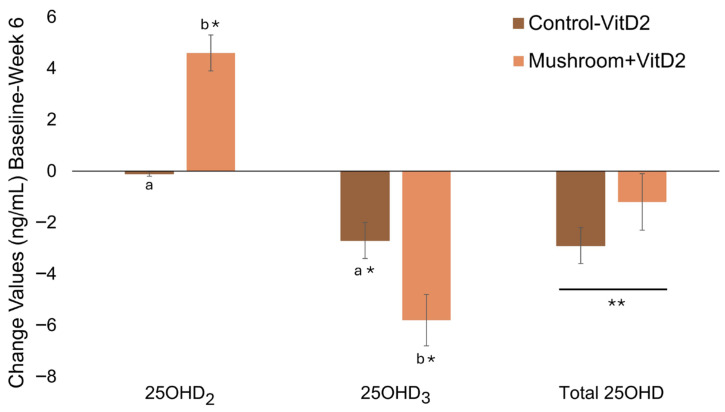
Post hoc analysis based on measured serum 25OHD_2_ concentrations. Change values (ng/mL; baseline to week 6) of 25OHD_2_, 25OHD_3_, and total 25OHD for participants whose sera showed detectable concentrations of 25OHD_2_ (>1.5 ng/mL; Mushroom + VitD_2_) and those whose sera did not (<1.5 ng/mL) combined with ITT Control (Control-VitD_2_). * Denotes significant change from baseline. Different letters (a, b) denote significant differences in the change values between groups. ** Denotes main effect of time in the absence of an interaction effect.

Based on this post hoc analysis, consuming UVMs increased 25OHD_2_ (Time × Group, *p* < 0.001) but did not maintain total 25OHD (Time × Group, *p* = 0.238) due to greater decreases in 25OHD_3_ (Time × Group, *p* = 0.018; Supplementary Material S1 Table S4). Total 25OHD decreased independently of mushroom consumption. The increase in 25OHD_2_ in the Mushroom + VitD_2_ group (4.6 ± 0.7 ng/mL, mean ± SE) differed from Control-VitD_2_ (−0.1 ± 0.1 ng/mL, *p* < 0.001) and the decrease in 25OHD_3_ was greater in the Mushroom + VitD_2_ group (−5.8 ± 1.0) compared to Control-VitD_2_ (−2.7 ± 0.7 ng/mL, *p* = 0.008). Although the increase in 25OHD_2_ was greater in this post hoc analysis (4.6 ± 0.7 ng/mL) compared to ITT (2.4 ± 0.5 ng/mL), the overall results were comparable to ITT results. The interaction (change values) and main effects are depicted in Figure 4 and summarized in Supplementary Material S1 Table S4.

### 3.6. Post Hoc Analysis of Participants Consuming Mushrooms with and Without Vitamin D_2_

In this post hoc analysis, we compared three subgroups: the nine participants in the Mushroom group whose circulating 25OHD_2_ was below the detection limit (<1.5 ng/mL, Mushroom-VitD_2_, *n* = 9), the 11 participants in the Mushroom group whose serum 25OH D_2_ was >1.5 ng/mL (Mushroom + VitD_2_, *n* = 11), and the participants in the ITT (Control, *n* = 21). This analysis allowed us to explore whether the impact of consuming mushrooms containing vitamin D_2_ on serum 25OHD_2_ and 25OHD_3_ is due to the vitamin D_2_ content alone or whether there is an impact of mushrooms on these outcomes independent of the added vitamin D_2_.

**Figure 5:**
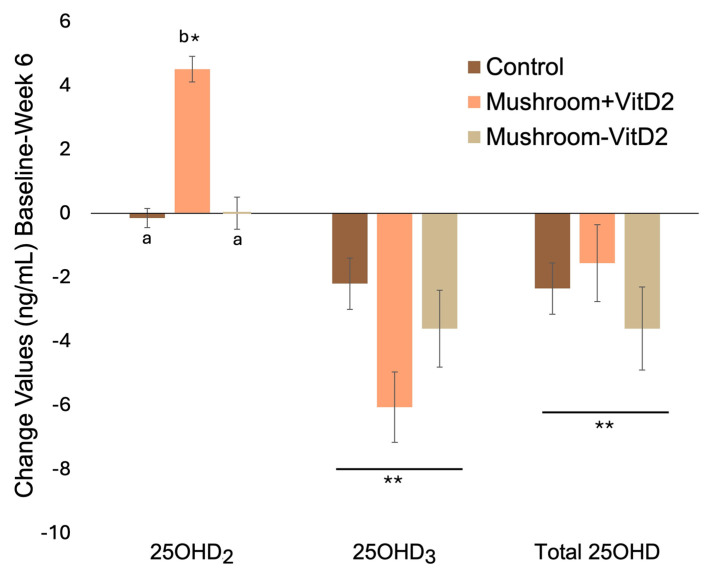
Post hoc analysis of participants consuming mushrooms with and without vitamin D_2_. Change values (ng/mL; baseline to week 6) of 25OHD_2_, 25OHD_3_, and total 25OHD for participants consuming D_2_-enriched mushrooms (Control, *n* = 21, vs. Mushroom-VitD_2_, *n* = 9, vs. Mushroom + VitD_2_, *n* = 11). * Denotes significant change from baseline. Letters (a, b) denote significant differences in change values between groups. ** Denotes main effect of time in the absence of interaction effect.

Based on this post hoc analysis (Control, *n* = 21, vs. Mushroom-VitD_2_, *n* = 9, vs. Mushroom + VitD_2_, *n* = 11)*,* consuming UVMs increased 25OHD_2_ (Time × Group, *p* < 0.001) but did not maintain total 25OHD (Time × Group, *p* = 0.357) due to decreases in 25OHD_3_ (Time × Group, *p* = 0.039; note *p*-value not significant after correcting for multiple comparisons (FDR 5%)). Total 25OHD decreased independently of mushroom consumption. The increase in 25OHD_2_ in the Mushroom + VitD_2_ group (4.6 ± 0.4 ng/mL, mean ± SE) differed from the Control (−0.2 ± 0.2 ng/mL, *p* < 0.001) and Mushroom-VitD_2_ (0.0 ± 0.4 ng/mL, *p* < 0.001) groups. The change in 25OHD_2_ between the Mushroom-VitD_2_ and the Control groups did not differ (*p* = 0.976). The decrease in 25OHD_3_ in the Mushroom + VitD_2_ group (−5.8 ± 1.0 ng/mL) was greater than the decrease in the Control (−2.3 ± 0.8 ng/mL; *p* = 0.031) but did not differ from the Mushroom-VitD_2_ (−3.7 ± 1.2 ng/mL, *p* = 0.480). The decrease in the Control did not differ from the Mushroom-VitD_2_ (*p* = 0.669). The interaction (change values) and main effects are depicted in Figure 5 and summarized in Supplementary Material S1 Table S5.

Based on planned and post hoc analyses, UVM consumption increased serum 25OHD_2_ but did not offset declines in total 25OHD during winter due to greater decreases in 25OHD_3_.

## 4. Discussion

To our knowledge, this study was the first to assess whether consuming commercially produced fresh UVMs would offset the seasonal decline in vitamin D status among adults classified as overweight and class I obese. Including a non-mushroom, non-vitamin D control allows a clear comparison of responses to the seasonal decline between people consuming mushrooms with vitamin D_2_ and those who are not consuming mushrooms (most U.S. adults [32]).

Five other studies have assessed vitamin D status using fresh UVMs. Four of the five (80%) assessed this outcome among people with normal BMI [12,13,18,33]. The one [15] that assessed this outcome among people with BMI > 25 kg/m^2^ did not include a non-mushroom, non-vitamin D control. The only study [13] that included a non-mushroom control (orange juice) assessed whether consuming UVMs containing a high vitamin D_2_ dose of 4000 IU/d would increase vitamin D status among healthy young adults. Three of the five (80% of) studies were conducted during winter months in high-latitude locations (≥40° N) where people are at risk of greater declines in vitamin D status [12,13,15]. One study [18] was conducted during summer through late fall (June–November) in a low-latitude location (<40° N) and another [33] conducted in a low-latitude location did not report the season. Vitamin D_2_ doses in the studies ranged from 500 IU/d [15] to 4000 IU/d [13].

Consistent with our hypothesis, UVM consumption increased serum 25OHD_2_. However, inconsistent with our hypothesis, UVM consumption did not maintain total 25OHD due to a greater decrease in 25OHD_3_. Results from this research are partly consistent with previous studies that investigated the effects of fresh UVM consumption on vitamin D metabolites. Three [13,18,33] of four [15] (75% of) studies reported increased 25OHD_2_, which is consistent with the results from the present study. Two (50%) [13,18] reported a decrease in 25OHD_3_, while the other two (50%) [15,33] that assessed this outcome reported no change. Two studies (50%) [12,13] reported an increase in total 25OHD while the other two (50%) [15,18] reported no change (i.e., maintained vitamin D status).

Looking at the broader UVM literature that includes powdered and encapsulated UVMs as the intervention, two systematic reviews [14,34] previously assessed the effects of consuming UVMs on 25OHD_2_, 25OHD_3_, and total 25OHD and both found that 25OHD_2_ increased, 25OHD_3_ decreased, and total 25OHD did not change. The first is a systematic review and meta-analysis by Cashman et al. [14] while the second is a systematic review only by Rondanelli et al. [34]. There was significant overlap of studies included (67%) in the two systematic reviews, but the systematic review by Rondanelli et al. included a large randomized controlled trial (*n* = 436) which nearly tripled the total number of participants involved.

Cashman et al. [14] conducted a systematic review and meta-analysis of six randomized controlled trials involving *n* = 244 participants. They reported that consuming UVMs increased 25OHD_2_ (random effect WMD: 8.3 ng/mL (20.6 nmol/L); 95% CI: 3.2, 13.3 ng/mL (8.0, 33.3 nmol/L); *p* < 0.001) and decreased 25OHD_3_ (random effect WMD: −5.3 ng/mL (−13.3 nmol/L); 95% CI: −6.3, −4.3 ng/mL (−15.8, −10.7 nmol/L); *p* < 0.00001). These results are consistent with the results from the present study. The authors also reported that UVM consumption maintained (did not change) total 25OHD (random effect WMD: 3.4 ng/mL; 95% CI: 8.9, 7.9 ng/mL; *p* = 0.12). This result is inconsistent with the present study due to the decrease in total 25OHD we observed.

Rondanelli et al. [34] conducted a systematic review of six randomized controlled trials involving *n* = 663 participants to assess the effects of UVM consumption on 25OHD_2_, 25OHD_3_, and total 25OHD, and other non-skeletal outcomes including cardiovascular, metabolic syndrome, and neuromuscular function. Most (4/6, 67% of) studies overlapped with the aforementioned systematic review and meta-analysis [14]. The authors found that UVM consumption increased 25OHD_2_, decreased 25OHD_3_, but did not change total 25OHD.

Consistent results from six [12,13,16,17,19,20] comparable studies (conducted during winter months, used whole UVMs, and had a non-vitamin D control) indicated that UVM consumption increased 25OHD_2_. However, results reporting on total 25OHD are mixed with 50% (3/6 studies [16,17,19]) reporting no changes, 33% (2/6 studies [12,13]) an increase, and 17% (1/6 study [20]) a decrease. All six studies were included in the systematic reviews by Cashman et al. [14] (2/6 studies) and Rondanelli et al. [34] (4/6 studies). Participants in high-latitude locations had lower baseline concentrations of total 25OHD [12,13], compared to those in low-latitude locations [16,17,20], potentially explaining the increase in total 25OHD after UVM consumption. Other factors that may have influenced the mixed results include different amounts of vitamin D_2_ in UVMs and groups of participants with different characteristics. One study [13] that reported an increase in total 25OHD fed participants UVMs containing five times the content of vitamin D_2_ (4000 IU/d) than in the present study (800 IU/d). Another study that reported a decrease [20] fed UVMs to a group of adults characterized as elderly, who may have had greater vitamin D needs.

Vitamin D_2_ is less effective at raising total 25OHD given its decreased hydroxylation and lower binding affinity for vitamin D binding protein [35,36]. Cytochrome P450 27A1 (CYP27A1) has lower binding affinity to vitamin D_2_ leading to less conversion to 25OHD_2_ [35]. Also, vitamin D_2_’s lower binding affinity for vitamin D binding protein may lead to its sequestration into fat cells and loss from degradation [35], thereby lowering total 25OHD in the blood. Additionally, total 25OHD decreases or does not change with vitamin D_2_ supplementation because of equal or greater declines in 25OHD_3_ [37,38,39]. With greater concentrations of vitamin D_2_ compared to D_3_ in circulation, CYP27A1, a liver enzyme, is competitively inhibited from hydroxylating vitamin D_3_, leading to reductions in 25OHD_3_. Possibly, increased degradation of 25OHD_3_ may also account for the reductions in 25OHD_3_ [14,19].

Beyond the exacerbated decrease in 25OHD3, the cause of the decrease in total 25OHD in the present study is not clear. It may be because people with overweight and obesity require greater daily doses of vitamin D_2_; because on average, participants in the present study were not deficient in vitamin D; or because vitamin D_2_ is less effective at raising total 25OHD. Some evidence suggests that people with overweight and obesity require two to three times the RDA for vitamin D to maintain total 25OHD [40] since adipose tissue can sequester vitamin D metabolites [35]. Following this logic, participants classified as overweight and obese should consume UVMs with 1200 to 1800 IU/d of vitamin D_2_. However, neither prior evidence nor our results support this hypothesis. To our knowledge, only two other randomized controlled clinical trials [15,19] assessing the effects of UVM consumption were conducted among adults classified as middle-aged and older with overweight and obesity. Both indicate that lower doses of vitamin D_2_ in UVMs (600 IU/d [19] and 500 IU/d [15]) were effective at offsetting declines in vitamin D status among adults with overweight and obesity during winter months. The results of the present study indicate that an average intake of 1120 IU/d of vitamin D_2_ does not offset the decline in vitamin D status among adults with overweight and obesity during winter months. Thus, the reason for the decrease in total 25OHD in the present study may not be solely explained by the amount of vitamin D_2_ intake.

It is well established that baseline concentrations of vitamin D predict the response to vitamin D supplementation. When baseline vitamin D status is low (<20 ng/mL), the increase in 25OHD_2_ is greater than the decrease in 25OHD_3_ resulting in an overall increase in vitamin D status [14]. Of the previous UVM studies, no study included participants with vitamin D deficiency. Two studies included participants with vitamin D insufficiency (<20 ng/mL) and both reported that UVM consumption offsets the decrease in total 25OHD during winter [13,17]. In the present study, participants consuming mushrooms were, on average, sufficient for vitamin D at baseline (total 25OHD of 21.7 ± 5.6 ng/mL; mean ± SD). The higher baseline concentrations of total 25OHD may explain a smaller response of 25OHD_2_ and therefore the overall decrease in vitamin D status.

Strengths of the present study include being conducted during winter months; using whole, fresh UVMs; having a non-mushroom, non-vitamin D control; and participants’ high adherence to the interventions (97% mean self-reported adherence) and a low drop-out percentage (<5%). Being conducted in Indiana, USA, during the winter months (late October to early April), the present study design diminished endogenous vitamin D_3_ synthesis from sun exposure. Furthermore, participants were asked to forego tanning procedures and vitamin D supplements during the study period. To minimize the risk of bias, we removed identifying information from the data, two researchers entered the data independently, and we verified consistency through cross-checking. A limitation of the present study includes the inability to blind research coordinators and participants in the Mushroom group due to using whole, fresh mushrooms as the intervention. Another limitation is that this study was powered on hypothetical changes in total 25OHD due to no prior studies with comparable study designs.

Future research powered to detect changes in total 25OHD using the variability we report is warranted to corroborate our findings. Additionally, the implications and reasons for the greater decrease in 25OHD_3_ are not clearly understood. Future studies should expand on work started by Stephensen et al. [18] and measure degradation metabolites in serum. Finally, future studies should explore the long-term implications of the greater decrease in 25OHD_3_ on classic (i.e., calcium homeostasis) and non-classic outcomes.

## 5. Conclusions

Consumption of commercially available D_2_-enriched mushrooms intended to deliver 800 IU of vitamin D_2_ per day as part of a habitual diet does not offset the seasonal decline in total 25OHD, an important biomarker of vitamin D status. Consuming D_2_-enriched mushrooms increased 25OHD_2_ concentrations and decreased 25OHD_3_. Since the decline in 25OHD_3_ was greater, vitamin D status was not maintained. The reason vitamin D_2_ in the mushrooms did not maintain total 25OHD is unclear but may be because of insufficient vitamin D_2_ in the mushrooms or because 25OHD_2_ has decreased hydroxylation and lower binding affinity for vitamin D binding protein compared to 25OHD_3_.

## Figures and Tables

**Table 1:** Intent-to-treat: Demographic and clinical characteristics of participants at baseline.

Demographic Characteristics	Control (*n* = 21)	Mushroom (*n* = 20)	Total (*n* = 41)
Age (y)	42.2 ± 11.2	44.0 ± 11.1	43.1 ± 11.1
Female, *n* (%)	12 (57)	10 (50)	22 (54)
White, *n* (%)	11 (52)	15 (75)	26 (63)
Hispanic or Latino, *n* (%)	5 (24)	2 (10)	7 (17)
Asian, *n* (%)	4 (19)	3 (15)	7 (17)
Black, *n* (%)	1 (5)	1 (5)	2 (5)
Other (not specified/not reported), *n* (%)	5 (24)	1 (5)	6 (15)
Weight (kg)	82.3 ± 10.0	87.3 ± 15.6	84.7 ± 13.1
BMI (kg/m^2^)	29.9 ± 7.9	29.7 ± 2.7	29.8 ± 5.9
Baseline HEI Score (au)	50 ± 15.3	55 ± 15.3	53 ± 15.4
**Fasted Clinical Characteristics**			
25OHD_3_ (ng/mL)	23.3 ± 7.5	21.7 ± 5.6	22.5 ± 6.6
25OHD_2_ (ng/mL)	0.2 ± 0.9	0.0 ± 0.0	0.1 ± 0.6
Total 25OHD (ng/mL) ^1^	23.5 ± 7.8	21.7 ± 5.6	22.6 ± 6.8
BUN (mg/dL)	15.1 ± 5.0	12.3 ± 3.0	13.7 ± 4.0
Creatinine (mg/dL)	0.9 ± 0.2	0.8 ± 0.1	0.8 ± 0.1
BUN/Creatinine Ratio	15.1 ± 5.0	15.1 ± 3.4	15.1 ± 4.3
eGFR (mL/min/1.73 m^2^)	98.1 ± 13.0	101.6 ± 13.3	99.7 ± 13.1
ALT (U/L)	19.1 ± 13.7	18.6 ± 9.4	18.9 ± 11.7
AST (U/L)	19.6 ± 7.3	20.1 ± 5.2	19.8 ± 6.3

Results are means ± SD unless otherwise indicated. ^1^ Total 25OHD = 25OHD_3_ + 25OHD_2_. Abbreviations: ALT, alanine transaminase; AST, aspartate transferase; BUN, blood urea nitrogen; eGFR, estimated glomerular filtration rate; HEI, Healthy Eating Index (arbitrary unit, au, range 0–100).

**Table 2:** Dietary intake of participants.

	Control	Mushroom	Total
Energy Intake (kcal/d)	1996 ± 831	1778 ± 574	1890 ± 717
Protein:Fat:Carbohydrate (% energy)	28:28:43	27:26:47	28:27:45
Dietary Vitamin D * (μg/d)	4.5 ± 0.9	4.9 ± 1.9	4.7 ± 1.5
Dietary Vitamin D * (IU/d)	180 ± 36	196 ± 76	188 ± 60

Data from the Automated Self-Administered 24 h Dietary Assessment Tool (NCI/NIH) were used for calculating results. Results are means ± SD unless otherwise indicated. Differences between groups were not statistically significant by an independent sample, two-tailed *t*-test. * Dietary vitamin D is D_2_ + D_3_.

**Table 3:** Healthy Eating Index scores of participants at baseline and during the intervention.

Timepoint	Control	Mushroom	Total
Baseline	55 ± 15.3	50 ± 15.3	53 ± 15.4
Intervention	52 ± 14.1	50 ± 10.9	51 ± 12.5
Baseline + Intervention	53 ± 12.1	50 ± 10.3	52 ± 11.2

Results are means ± SD. Heathy Eating Index scores (HEI) can range from 0 to 100 arbitrary units, with a higher score indicating a healthier dietary pattern [24]. HEI scores at the intervention and baseline + intervention timepoints were not statistically different between groups. Table reproduced from Glover et al. 2025 [21].

## Data Availability

The original contributions presented in this study are included in the article/Supplementary Materials. Further inquiries can be directed to the corresponding author.

## Supplementary Materials

The following supporting information can be downloaded at https://www.mdpi.com/article/10.3390/foods15091572/s1.
