# Supplementary material for: Does Consuming Fresh Ultraviolet Light-Exposed Mushrooms Offset the Seasonal Decline in Serum Total 25OHD in Adults Classified as Overweight and Class I Obese? Results from a Randomized Controlled Trial
Source: Foods. 2026 May 2;15(9):1572. doi: 10.3390/foods15091572 (PMC13163831; doi:10.3390/foods15091572)
Supplement: Supplementary file 1 [file foods-15-01572-s001.zip › Supplementary Material S1 FINAL.pdf]

Supplementary Material S1

Does Consuming Fresh Ultraviolet Light-Exposed Mushrooms Offset the Seasonal Decline in Serum Total 25OHD in Adults Classified as Overweight and Class I Obese? Results from a Randomized Controlled Trial – Comboni LM & Glover ES et al.

Supplementary Table S1: Inclusion and Exclusion Criteria

**Inclusion Criteria**	• BMI: 25.0-34.9 kg/m^2<br>• Ages 30-69 years<br>• total cholesterol < 240 mg/dL<br>• low density lipoprotein cholesterol < 160 mg/dL<br>• triglycerides < 300 mg/dL<br>• fasting glucose < 110 mg/dL<br>• systolic/diastolic blood pressure < 140/90 mmHg<br>• body weight stable for 3 months prior (± 3 kg)<br>• stable physical activity regiment 3 months prior<br>• medication use stable for 6 months prior<br>• non-smoking<br>• non-diabetic<br>• not acutely ill<br>• females not pregnant or lactating<br>• willing to refrain from taking vitamin D supplements or any supplements containing vitamin D<br>• No history of bariatric surgery<br>• Not extremely or severely depressed (Beck's Depression Inventory Score ≤ 30)<br>• Agree not to donate blood for at least one month prior to, during, and for one month after the study<br>• Agree not to travel to sunny locations during the study period<br>• Agree to forgo any tanning bed or other tanning procedures during the study<br>• Willing and able to consume mushrooms and travel to testing facilities
**Exclusion Criteria**	• Regimen of mega-dosing vitamin D (> 3000 IU/d)<br>• History of bariatric surgery<br>• Consuming a restricted diet (such as keto, gluten-free, high protein, low carb, etc.)<br>• Allergic to mushrooms or bread

Supplementary Material S1

Does Consuming Fresh Ultraviolet Light-Exposed Mushrooms Offset the Seasonal Decline in Serum Total 25OHD in Adults Classified as Overweight and Class I Obese? Results from a Randomized Controlled Trial – Comboni LM & Glover ES et al.

Supplemental Table S2. *Intent-to-treat*: Unadjusted means, SD, sample size, and Cohen's d effect size by outcome

	**Mushroom**									**Control**									Cohen's d[1]
	Baseline			Week 6			Change			Baseline			Week 6			Change			
Outcome	Mean	SD	*n*	Mean	SD	*n*	Mean	SD	*n*	Mean	SD	*n*	Mean	SD	*n*	Mean	SD	*n*	
$25OHD_2$ (ng/mL)	0.0	0.0	20	2.52	2.83	20	2.52	2.83	20	0.19	0.87	21	0.0	0.0	21	-0.19	0.87	21	-1.31
$25OHD_3$ (ng/mL)	21.66	5.55	20	16.78	4.20	20	-4.88	3.21	20	23.26	7.54	21	20.97	7.08	21	-2.29	3.83	21	0.73
Total 25OHD (ng/mL)	21.66	5.55	20	19.30	4.78	20	-2.36	3.55	20	23.45	7.84	21	20.97	7.08	21	-2.48	4.10	21	-0.03

[1]Cohen's d effect size is estimated using the mean change values (Mushroom-Control)

Supplementary Material S1

Does Consuming Fresh Ultraviolet Light-Exposed Mushrooms Offset the Seasonal Decline in Serum Total 25OHD in Adults Classified as Overweight and Class I Obese? Results from a Randomized Controlled Trial – Comboni LM & Glover ES et al.

Supplementary Table S3. *Intent-to-treat*: Effects of consuming UV light-exposed mushrooms or control for 6 weeks on serum vitamin D metabolites

Outcome (ng/mL)	Control (n=21)			Mushroom (n=20)			p-Values		
	Baseline	Week 6	Change	Baseline	Week 6	Change	Time	Group	Time × Group
$25OHD_2$	0.2 ± 0.1	0.1 ± 0.4	0.1 ± 0.5	0.1 ± 0.1	2.5 ± 0.4	2.4 ± 0.5	< 0.001	< 0.001	< 0.001
$25OHD_3$	23.2 ± 1.5	20.9 ± 1.3	-2.3 ± 0.8	21.7 ± 1.5	16.8 ± 1.3	-4.9 ± 0.8	< 0.001	0.149	0.033
Total 25OHD	23.4 ± 1.5	20.9 ± 1.3	-2.5 ± 0.9	21.7 ± 1.5	19.4 ± 1.4	-2.3 ± 0.9	0.008	0.459	0.910

Results are mean ± SE. $25OHD_2$, $25OHD_3$ analyzed by LC/MS/MS. Total 25OHD = $25OHD_2$ + $25OHD_3$.

Supplementary Table S4. *Post hoc* analysis based on measured serum $25OHD_2$ concentrations

Outcome (ng/mL)	Control-VitD_2 (n=30)			Mushroom+VitD_2 (n=11)			p-Values		
	Baseline	Week 6	Change	Baseline	Week 6	Change	Time	Group	Time × Group
$25OHD_2$	0.1 ± 0.1	0.0 ± 0.2	-0.1 ± 0.1	0.0 ± 0.2	4.6 ± 0.3	4.6 ± 0.7	< 0.001	< 0.001	< 0.001
$25OHD_3$	22.6 ± 1.2	19.9 ± 1.1	-2.7 ± 0.7	22.1 ± 2.0	16.2 ± 1.8	-5.8 ± 1.0	< 0.001	0.337	0.018
Total 25OHD	22.8 ± 1.3	19.9 ± 1.1	-2.9 ± 0.7	22.1 ± 2.1	20.9 ± 1.9	-1.2 ± 1.1	0.022	0.949	0.238

Results are mean ± SE. $25OHD_2$, $25OHD_3$ analyzed by LC/MS/MS. Total 25OHD = $25OHD_2$ + $25OHD_3$.

Supplementary Table S5. *Post hoc* analysis of subjects consuming UV light-exposed and non-UV light-exposed mushrooms

Outcome (ng/mL)	Control (n=21)			Mushroom-VitD_2 (n=9)			Mushroom+VitD_2 (n=11)			p-Values		
	Baseline	Week 6	Change	Baseline	Week 6	Change	Baseline	Week 6	Change	Time	Group	Time × Group
$25OHD_2$	0.2 ± 0.1	0.0 ± 0.2	-0.2 ± 0.2	0.0 ± 0.2	0.0 ± 0.4	0.0 ± 0.4	0.0 ± 0.2	4.6 ± 0.3	4.6 ± 0.4	< 0.001	< 0.001	< 0.001
$25OHD_3$	23.2 ± 1.5	20.9 ± 1.3	-2.3 ± 0.8	21.2 ± 2.3	17.5 ± 2.0	-3.7 ± 1.2	22.1 ± 2.1	16.3 ± 1.8	-5.8 ± 1.0	< 0.001	0.357	0.039*
Total 25OHD	23.4 ± 1.5	20.9 ± 1.3	-2.5 ± 0.8	21.2 ± 2.4	17.4 ± 2.1	-3.7 ± 1.3	22.1 ± 2.1	20.9 ±1.8	-1.2 ± 1.2	< 0.001	0.532	0.357

Results are mean ± SE. * p-value = 0.039 is not significant after correction for multiple comparisons (False Discovery Rate: 5%). $25OHD_2$, $25OHD_3$ analyzed by LC/MS/MS. Total 25OHD = $25OHD_2$ + $25OHD_3$.
