# Supplementary material for: Does Consuming Fresh Ultraviolet Light-Exposed Mushrooms Offset the Seasonal Decline in Serum Total 25OHD in Adults Classified as Overweight and Class I Obese? Results from a Randomized Controlled Trial
Source: Foods. 2026 May 2;15(9):1572. doi: 10.3390/foods15091572 (PMC13163831; doi:10.3390/foods15091572)
Supplement: Supplementary file 1 [file foods-15-01572-s001.zip › Supplementary Material S2 FINAL.pdf]

Supplementary Material S2

Does Consuming Fresh Ultraviolet Light-Exposed Mushrooms Offset the Seasonal Decline in Serum Total 25OHD in Adults Classified as Overweight and Class I Obese? Results from a Randomized Controlled Trial – Comboni LM & Glover ES et al.

Power Calculations

To our knowledge, this is the first study to answer the question, "does consuming ultraviolet light-exposed mushrooms (UVM) offset the seasonal decline in vitamin D status during winter compared to consuming a non-mushroom control among adults classified as overweight and class I obese?" As such, no preliminary data was available for power calculations. Retrospectively, we estimated the sample size required for 80% power (1-β) at α=0.05 based on our hypothesis and assuming a 30% wintertime decline in total 25OHD. Below we describe the retrospective power calculations based on our hypothesis and based on the observed differences and variability in this study.

Based on our hypothesis– Consuming UVM would increase $25OHD_2$ and offset the seasonal decline in total 25OHD. That is, there would be a difference between groups (δ) for $25OHD_2$, no difference for $25OHD_3$, and the same difference for total 25OHD that was observed for $25OHD_2$. Assuming a wintertime decline of 30% in vitamin D status and a starting value of 20ng/mL of total 25OHD, we estimated n=8 per group to detect a change of δ = 6 ng/mL (Table S1). With a sample size n=20 per group, we have power to detect a change of 3.3 ng/mL in serum total 25OHD.

Table S1. Numbers used to estimate sample size (n) per group.

Metabolite	SD (ng/mL)	δ (ng/mL)	Estimated sample size (n) per group
$25OHD_2$	1.33	6	3
$25OHD_3$	3.67	0.1	21,145
Total 25OHD	3.98	6	8

Calculations were performed using the proc power function in SAS Studio v. 2025.09. SD and δ are observed variability and differences in the present study. δ = mean difference.

Based on observed differences between participants with detectable serum concentrations of $25OHD_2$ (n=11) compared to those in the non-mushroom control (n=30) with no detectable serum $25OHD_2$– There was a differential response between groups for $25OHD_2$ and $25OHD_3$, but no difference in total 25OHD (Table S2). These calculations could be used to power future studies with a similar study design.

Table S2. Numbers that can be used to estimate future sample size (n) per group.

Metabolite	SD (ng/mL)	δ (ng/mL)	Estimated sample size (n) per group
$25OHD_2$	1.33	4.5	3
$25OHD_3$	3.67	3.1	19
Total 25OHD	3.98	1.7	88

Calculations were performed using the proc power function in SAS Studio v. 2025.09. SD and δ are observed variability and differences in the present study. δ = mean difference.
