# Supplementary material for: Does Consuming Fresh Ultraviolet Light-Exposed Mushrooms Offset the Seasonal Decline in Serum Total 25OHD in Adults Classified as Overweight and Class I Obese? Results from a Randomized Controlled Trial
Source: Foods. 2026 May 2;15(9):1572. doi: 10.3390/foods15091572 (PMC13163831; doi:10.3390/foods15091572)
Supplement: Supplementary file 1 [file foods-15-01572-s001.zip › Supplementary Material S3 FINAL.pdf]

Supplementary Material S3 – Report and discussion of the variability of the vitamin D_2 content in the mushrooms

Does Consuming Fresh Ultraviolet Light-Exposed Mushrooms Offset the Seasonal Decline in Serum Total 25OHD in Adults Classified as Overweight and Class I Obese? Results from a Randomized Controlled Trial – Comboni LM & Glover ES et al.

How variable was the vitamin D_2 intake of participants assigned to consume mushrooms (intent to treat; n=20)?

There was high variability of vitamin D_2 intake among participants assigned to consume mushrooms (SD=119; CV=70%; Table 1). Using the vitamin D_2 content in mushroom samples as an intake proxy, we can estimate participant's average weekly vitamin D_2 intake and its variability. We first determined each participant's median intake from the vitamin D_2 content of mushroom samples analyzed weekly (n=6 total) during their intervention period. We estimated missing values by averaging the vitamin D_2 values from the preceding and following weeks. The mean vitamin D_2 content among these participants was calculated as the average value among 20 medians.

Table S1. Descriptives of vitamin D2 content (ng/g) in frozen mushroom samples

	n	Mean †	SD	CV	µg/d *	IU/d *
Vitamin D_2 (ng/g) in mushrooms	20	169	119	70%	28	1120

† Due to high variability in vitamin D_2 content among the mushroom samples, the mean intake of the group of participants who were assigned to consume mushrooms was estimated by first determining each participant's median intake from the vitamin D_2 content of mushroom samples analyzed weekly (n=6 total) during their intervention period. We estimated missing values by averaging the vitamin D_2 values from the preceding and following weeks. The mean vitamin D_2 content among the group of participants in the mushroom group was calculated as the average value among the 20 medians. * Values were calculated using the mean (169 ng/g). Abbreviations: CV: Coefficient of variation; SD: Standard deviation; IU: International Unit. Each 84-g sample of mushrooms collected during the intervention periods was homogenized and 1 g of the homogenous mixture was aliquoted for vitamin D_2 analysis using LC/MS/MS. Analyses were done at Heartland Assays, Ames IA.

How variable was the vitamin D_2 intake of participants that received mushrooms with vitamin D_2 (n=11)?

There was high variability of vitamin D_2 intake among participants that received mushrooms with vitamin D_2 (SD=97; CV=44%; Table 2). Participants whose serum showed $25OHD_2$ at week 6 (n=11), participated in 2022-2023 (n=3) and most in 2023-2024 (n=8). We know the vitamin D_2 content in mushrooms during this timeframe. Using the vitamin D_2 content in mushroom as an intake proxy, we can estimate the average vitamin D_2 intake of these participants and its variability. We first determined each participant's median intake from the vitamin D_2 content of mushroom samples analyzed weekly (n=6 total) during their intervention period. We estimated missing values by averaging the vitamin D_2 values from the preceding and following weeks. The mean vitamin D_2 content among these participants was calculated as the average value among 11 medians.

Table S2. Descriptives of vitamin D2 content in mushroom samples that had measurable vitamin D_2

Supplementary Material S3 – Report and discussion of the variability of the vitamin D_2 content in the mushrooms

Does Consuming Fresh Ultraviolet Light-Exposed Mushrooms Offset the Seasonal Decline in Serum Total 25OHD in Adults Classified as Overweight and Class I Obese? Results from a Randomized Controlled Trial – Comboni LM & Glover ES et al.

	n	**Mean †**	**SD**	**CV**	**μg/d ***	**IU/d ***
Vitamin D_2 (ng/g) in mushrooms	11	220	97	44%	37	1480

† To estimate the mean vitamin D_2 content of the mushroom samples that were UV exposed, we calculated the mean intake of the participants whose serum showed $25OHD_2$ at week 6 testing. We first determined each participant's median intake from the vitamin D_2 content of mushroom samples analyzed weekly (n=6 total) during their intervention period. We estimated missing values by averaging the vitamin D_2 values from the preceding and following weeks. The mean vitamin D_2 content among the group of participants in the mushroom group was calculated as the average value among the 11 medians. * Values were calculated using the mean (220). Abbreviations: CV: coefficient of variation; SD: standard deviation; IU: International Unit. Each 84-g sample of mushrooms collected during the intervention periods was homogenized and 1 g of the homogenous mixture was aliquoted for vitamin D_2 analysis using LC/MS/MS. Analyses were done at Heartland Assays, Ames IA.

Is this evidence convincing that the vitamin D_2 intake for participants with verified $25OHD_2$ was highly variable?

Yes, the evidence supports that the average weekly vitamin D_2 intake of participants was highly variable. The strength of this analysis is that the estimate is statistically reliable because we considered the skewness of the data by using the median as the estimator of each participant's vitamin D_2 intake during their intervention. However, this analysis is limited to weekly, not daily, vitamin D_2 intake since we were unable to sample mushrooms daily.

Was the lack of $25OHD_2$ at week 6 in nine participants assigned to consume mushrooms due to not eating the mushrooms or no vitamin D_2 content in the mushrooms?

The lack of $25OHD_2$ at week 6 in five of the nine participants was due to consuming mushrooms without vitamin D_2. We plotted two figures: 1) a horizontal bar graph of the intervention lengths of participants assigned to consume mushrooms with their serum $25OHD_2$ concentration at week 6 shown at the end of each bar (Figure 1), and 2) a scatter plot of the vitamin D_2 content in mushroom samples collected throughout the enrollment periods (Figure 2). Juxtaposing Figures 1 and 2, we observe that five instances of 0 ng/mL of $25OHD_2$ at week 6 overlap with < 6 ng/g of vitamin D_2 in the mushrooms in January 2024 (see vertical blue lines). Meaning, five of the nine participants showing no $25OHD_2$ in their sera at week 6 consumed mushrooms without vitamin D_2. It is difficult to ascertain the reason for the lack of $25OHD_2$ in the sera of the other four participants. They may have been non-adherent to the intervention, despite their self-reported adherence, or may have had decreased absorption or metabolism of vitamin D_2. Of note, one of these four participants reported allergies to apples, and another reported having problems eating high-fat foods.

Supplementary Material S3 – Report and discussion of the variability of the vitamin D_2 content in the mushrooms

Does Consuming Fresh Ultraviolet Light-Exposed Mushrooms Offset the Seasonal Decline in Serum Total 25OHD in Adults Classified as Overweight and Class I Obese? Results from a Randomized Controlled Trial – Comboni LM & Glover ES et al.

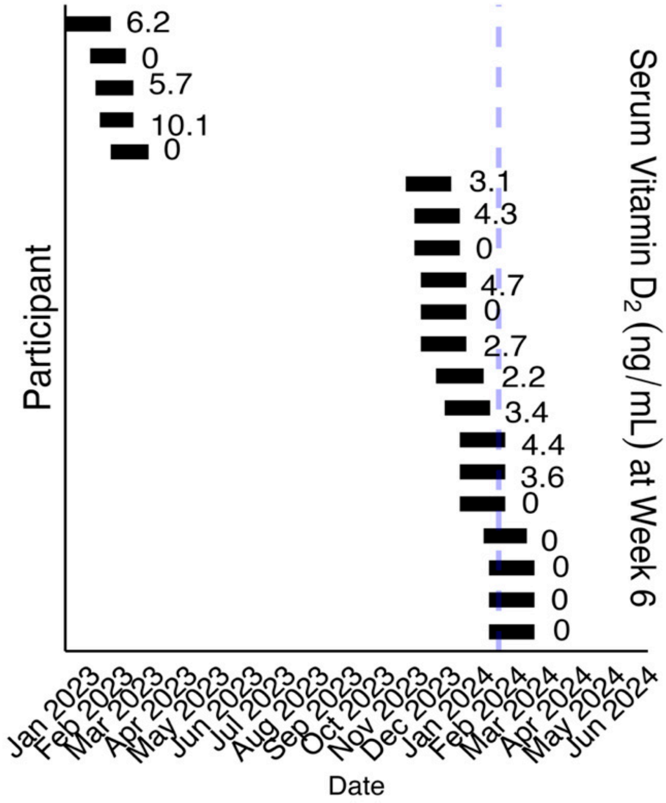

Figure S1. Chronological intervention lengths and serum vitamin D_2 at week 6 of each participant consuming mushrooms**.** Each horizontal bar graph represents the 6-week intervention for one participant, and their corresponding serum vitamin D_2 at week 6 is displayed next to it. The vertical line indicates the date when vitamin D_2 content in the mushroom consistently began to measure 0 ng/g.

Supplementary Material S3 – Report and discussion of the variability of the vitamin D_2 content in the mushrooms

Does Consuming Fresh Ultraviolet Light-Exposed Mushrooms Offset the Seasonal Decline in Serum Total 25OHD in Adults Classified as Overweight and Class I Obese? Results from a Randomized Controlled Trial – Comboni LM & Glover ES et al.

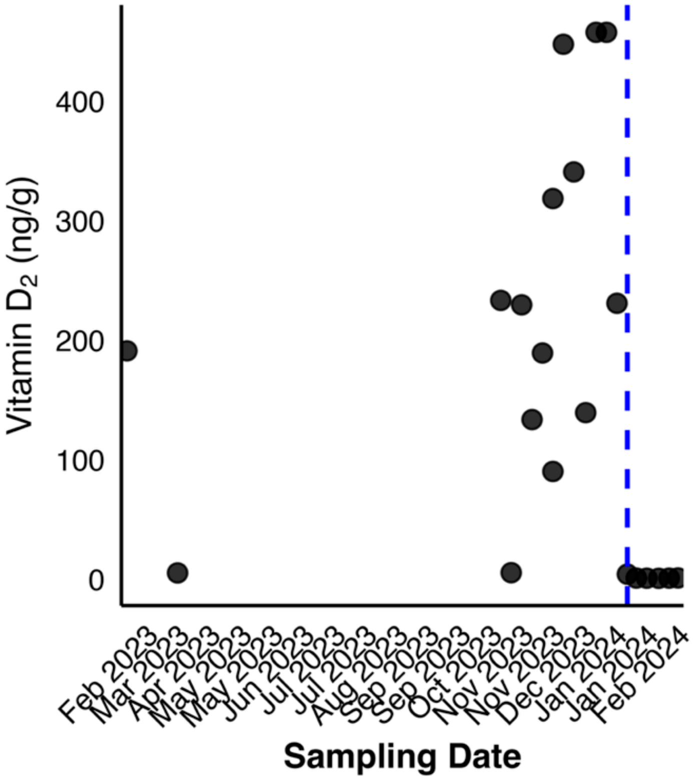

Figure S2. Vitamin D_2 content in mushrooms during both enrollment periods (2022-2023 and 2023-2024). The vertical, dashed line represents the date when mushroom samples consistently began to measure 0 ng/g of vitamin D_2.
